# Design and implementation of a basic and global point of care ultrasound (POCUS) certification curriculum for emergency medicine faculty

**DOI:** 10.1186/s13089-022-00260-y

**Published:** 2022-02-19

**Authors:** Frances M. Russell, Sarah K. Kennedy, Loren K. Rood, Benjamin Nti, Audrey Herbert, Matt A. Rutz, Megan Palmer, Robinson M. Ferre

**Affiliations:** Department of Emergency Medicine, Indiana University School of Medicine, 720 Eskenazi Ave, Fifth Third Faculty Office Building, 3rd Floor Emergency Medicine Office, Indianapolis, IN 46202 USA

**Keywords:** Point of Care Ultrasound, Medical Education, Emergency Medicine Faculty, Implementation, Credentialing

## Abstract

Point of care ultrasound (POCUS) use in the emergency department is associated with improved patient outcomes and increased patient satisfaction. When used for procedural guidance, it has been shown to increase first pass success and decrease complications. As of 2012, ultrasound has been identified as a core skill required for graduating emergency medicine (EM) residents. Despite this, only a minority of EM faculty who trained prior to 2008 are credentialed in POCUS. Half of all EM training programs in the United States have less than 50% of their faculty credentialed to perform and teach POCUS to learners. As the use of POCUS continues to grow in medicine, it is especially important to have a pathway for faculty to attain competence and become credentialed in POCUS. The goal of this paper was to outline an implementation process of a curriculum designed to credential EM faculty in POCUS.

**Supplementary Information:**

The online version contains supplementary material available at 10.1186/s13089-022-00260-y.

## Background

Emergency Physicians have been incorporating point of care ultrasound (POCUS) into the clinical care of patients for over 2 decades [1]. Guidelines for POCUS use, training and credentialing of Emergency Physicians were first published by the American College of Emergency Physicians in 2001 and have since been updated on two separate occasions to reflect the increased use and growth of POCUS within the clinical practice of emergency medicine. For a decade, POCUS has been a core competency of emergency medicine training and competency assessment is mandated by the Residency Review Committee of the Accreditation Council for General Medical Education [2, 3]. However, this growth in POCUS use and training has left many emergency physicians who trained before POCUS was widely used or mandated behind their peers. This training gap is pervasive throughout emergency departments today, even those with emergency medicine residency and advanced POCUS fellowship training programs [3].

There is a wealth of data showing the utility of POCUS for common patient presentations in emergency medicine. These data demonstrate that POCUS use is more accurate than traditional diagnostic tests, saves time and improves efficiency of care [4–8]. Additionally, POCUS is associated with fewer complications when used to guide invasive procedures [9]. Despite these benefits, practicing physicians who were never trained or became proficient in POCUS struggle to achieve the necessary training once residency is complete. For practicing clinicians, learning a new skill can be challenging. Lack of time, equipment and training, are the most commonly cited barriers to using POCUS in practice [10–12]. Objections by the Department of Radiology and lack of a formal credentialing process can also pose significant challenges [13].

Descriptions of POCUS training programs for practicing emergency physicians have previously been reported [13–15]. Smalley et al. [14] implemented a successful training curriculum for 106 community emergency physicians across a hospital system. Budhram et al. [13] found a step-wise incentive-based approach to be successful in a small cohort of academic emergency physicians. Cormack et al. [15] used a collaborative training approach with the radiology department to train 96 emergency physicians. These manuscripts focus primarily on outcomes and lack descriptive detail needed to replicate the curriculum. To our knowledge, no previous descriptions exist for a large mixed academic and community emergency department. The purpose of this paper is to describe the experience of a large, multi-site, mixed academic and community emergency department in creating and implementing a POCUS training and credentialing pathway for practicing emergency physicians.

## Curriculum

**Fig. 1:**
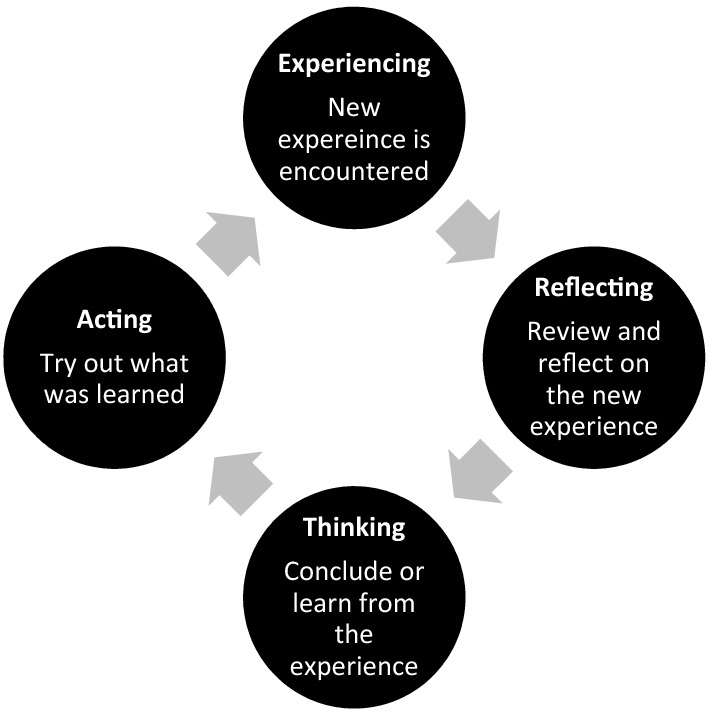
Kolb’s educational theory

Learning to perform and utilize POCUS within the clinical care of patients is a complex process. The objectives of the curriculum were based on four previously described sub-competencies: (1) understanding clinical indications for use; (2) developing technical skill for image acquisition; (3) interpreting images and (4) applying ultrasound findings clinically [16]. The curriculum was designed to be experiential in nature. Experiential learning theory, which is based on constructivist theories, posits that learning happens through experience. Kolb's experiential learning theory is represented by a four-stage learning cycle, see Fig. 1 [17].

There are numerous benefits to experiential learning, which include the opportunity to immediately apply knowledge, the promotion of team, increased motivation for learning, opportunity for reflection and real-world practice, which is why our curriculum was delivered in this manner. Furthermore, other emergency medicine scholars have examined the use of experiential learning in US education [18–20] and reported that learners believed that it improved their ability in the specific area of focus and that the experiential component was valuable to their learning.

The American College of Emergency Physicians (ACEP) outlines two pathways for ultrasound credentialing: residency- or practice-based pathway [1]. For the residency-based pathway, faculty had to provide proof of training within residency (a minimum of 150 scans as well as didactics) in the form of a standardized POCUS credentialing letter from their residency program. Physicians undergoing credentialing through the practice-based pathway were required to have a minimum of 16 h of continuing medical education (CME) and a minimum of 25 ultrasound exams. These examinations had to either be performed in the presence of another emergency physician with advanced POCUS training, or the study had to be documented, reviewed and deemed technically sufficient with an accurate interpretation during quality assurance (QA) review. Examinations could be performed on standardized patients or patients in the clinical environment. We did not require a minimum of abnormal examinations.

**Fig. 2:**
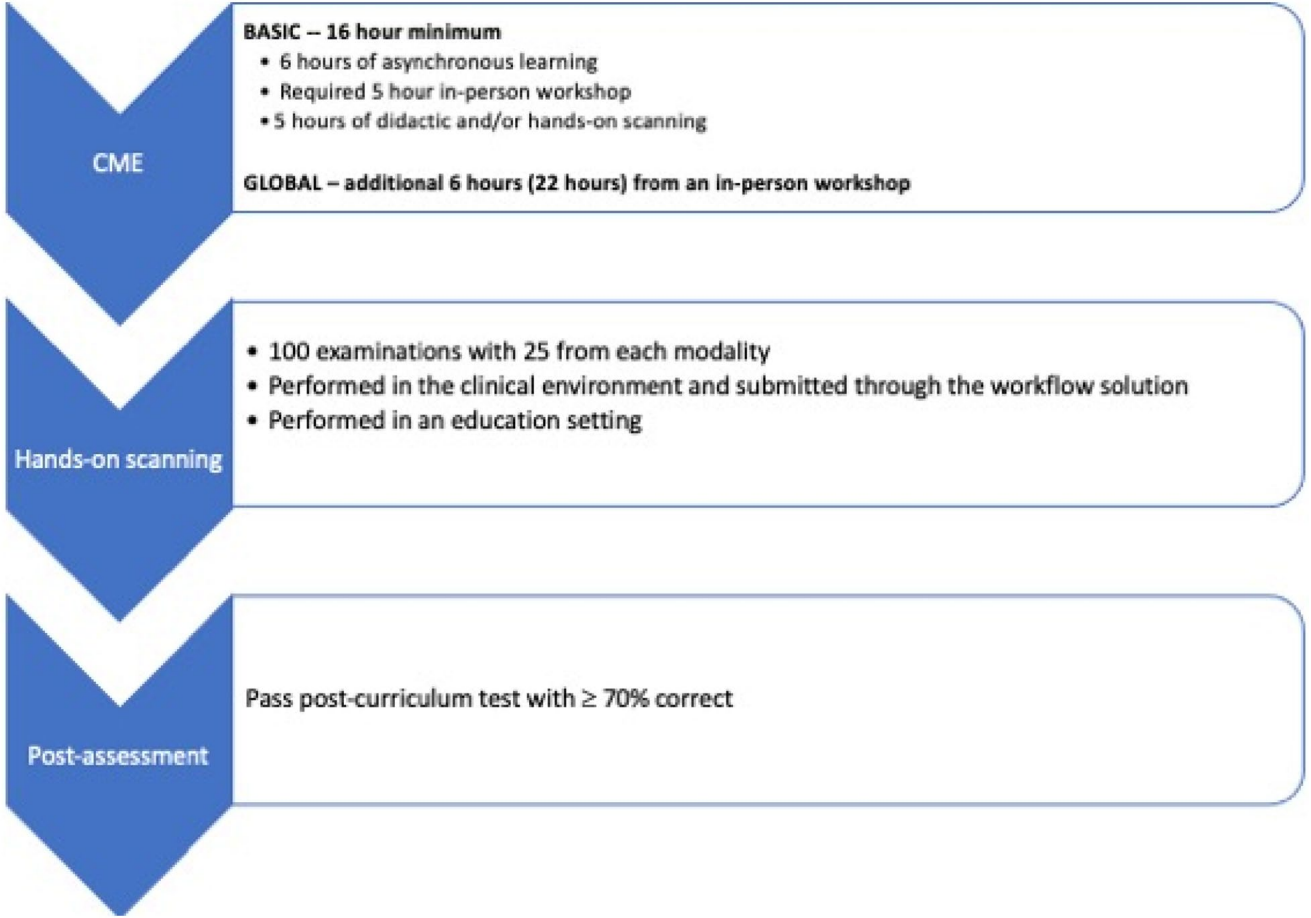
Flow of faculty through curriculum requirements

Due to the size and varied experience of practicing physicians in our department, the process of learning POCUS and meeting credentialing guidelines was envisioned as a multi-year process. Therefore, we created a two-tiered certification process that was designed as a step-wise approach to learning and achieving POCUS proficiency across an array of commonly performed POCUS exams. These two-tiers consisted of first achieving Basic Certification and then secondly, Global Certification in POCUS, see Table 1 for requirements and Fig. 1 for flow through curriculum (Fig. 2).

**Table 1:** Basic and global credentialing requirements

	CME (hours)	Examinations	Post-curriculum test
Basic	16	25 cardiac, 25 E-FAST, 25 aorta, 25 obstetric	50-question test, ≥ 70%correct
PEM	16	25 cardiac, 25 E-FAST, 25 thoracic, 25 soft tissue	50-question test, ≥ 70%correct
Global	Additional 6 (22 total)	Additional 100 (200 total) ocular, renal, thoracic, gallbladder, soft tissue, IV access, DVT, advanced cardiac, pediatric abdomen, musculoskeletal, regional nerve blocks	N/A

*CME* continuing medical education, *DVT* deep venous thrombosis, *E-FAST* extended focused sonography in trauma, *IV* intravenous, *PEM* pediatric emergency medicine

The pediatric emergency medicine (PEM)-specific POCUS certification curriculum was modeled after both the ACEP POCUS guidelines and the AAP policy statement on POCUS [1, 21]. In our curriculum, PEM-only faculty had the same CME requirements, however, the core applications differed. Instead of performing obstetric and aorta ultrasound, soft tissue and thoracic ultrasound were required. These examinations were selected based on PEM-specific applications common to the pediatric emergency department [22].

This curriculum was initially implemented in 2016 in the academic setting and expanded to community and pediatric sites in a staggard approach from 2018 to 2020. From implementation to December 2020, 176 emergency medicine faculty from 10 different hospitals have been trained: 1 pediatric tertiary academic hospital, 2 large, urban, tertiary academic hospitals, and 7 community hospitals. One hundred and forty-five faculty (82.4%) have achieved Basic Certification and 31 (17.6%) have not. Eighty-two faculty (46.9%) were trained via the residency-based pathway and 9 (5.1%) were ultrasound fellowship trained. Eighty-six of 176 faculty (48.9%) were on the practice pathway for certification. These faculty completed a total of 11,246 quality assured POCUS examinations for basic credentialing; 7849 were submitted and quality assured through the workflow solution and 3404 were from hands-on scanning sessions outside of the clinical environment. Sixty-five faculty (36.9%) have achieved Global Certification. There was no set timeline for completion of the curriculum, although the majority of faculty completed it within 2 years.

### Continuing medical education

For the initial 16 h of CME for Basic Certification, a maximum of 6 h of asynchronous POCUS learning was permitted. Faculty were provided links to online material including an online POCUS course that was developed for the curriculum, which consisted of physics, E-FAST, abdominal aorta, cardiac, renal, lung, and vascular access ultrasound modules. Additional information included instructions on how to use the workflow solution to submit examinations for QA. Other links provided included free open access material: SAEM narrated lecture series, Ultrasound Case of the Month, 5 Min Sono, Ultrasound Podcast, Sonoguide, The POCUS Atlas and the Ultrasound GEL Podcast.

At least 5 h of CME had to come from an in-person POCUS workshop with hands-on scanning. These CME events were taught by faculty within the department who were fellowship training in Emergency Ultrasound. These courses were offered several times a year at no cost to active faculty within the department. Additionally, physicians were also allowed to obtain this training through an external CME POCUS course.

The last 5 h of CME training allowed didactic and/or hands-on training that could be obtained from a variety of POCUS events offered within the department. These included: additional ultrasound workshops, ultrasound journal club, POCUS Grand Round lectures, participating in biweekly “Ultrasound Academic” sessions, scanning one-on-one with ultrasound faculty or from a “Drop in and sound” event. “Ultrasound Academic” sessions included ultrasound QA review and journal club led by ultrasound faculty.

The additional 6 h of CME required for Global Certification had to be from an in-person hands-on training workshop. This could include an internal advanced workshop, which covered more advanced topics than those taught during the basic workshop or an external course if similar topics were covered.

### Workshops

The internal basic ultrasound workshop included didactics and hands-on scanning. Didactics covered the core areas of aorta, cardiac, E-FAST, and obstetric applications as well as physics, knobology, the credentialing pathway, and instruction on how to use the workflow solution and submit exams for QA review. These methods have been previously described [10].

The first basic workshop was held in 2016 and has been given several times per year, each year since. This ensured appropriate level of initial training for all emergency physicians given the staggered approach to training within the department. Two advanced workshops were offered and covered different material. The first advanced workshop covered ocular, renal, thoracic, gallbladder, soft tissue and peripheral IV access. The second advanced workshop covered deep venous thrombosis, advanced cardiac, pediatric abdomen, musculoskeletal and regional nerve blocks. Simulated patients and phantom models were provided for learners to practice procedures.

### Hands-on scanning

Supervised hands-on training is a key component of POCUS education. Repetition allows the learner to develop the hand–eye coordination necessary for image acquisition. Accurate POCUS interpretation requires adequate imaging and structure visualization. Understanding how the various subtle ultrasound probe movements (rotating, sweeping, heel-toe rocking) can affect image acquisition and is best reinforced with repetition. Hands-on scanning also emphasizes proper machine adjustment techniques to optimize imaging. A variety of hands-on learning opportunities were offered throughout the curriculum in addition to the CME workshop and included “Drop in and sound” sessions and one-on-one supervised scanning sessions in the emergency department.

“Drop in and sound” sessions provided learners the opportunity to practice POCUS in a controlled setting. Scanning was performed on standardized patients and learners could rotate between stations with a 1:1 learner to POCUS faculty ratio. These sessions allow learners to ask questions, work on examinations they found difficult and build muscle memory for scan types through repetition.

One-on-one scanning sessions were arranged at the convenience of learner and instructor. These sessions were typically in local emergency departments with scans being performed on patients. These sessions provided an opportunity to review image acquisition, anatomy and pathology.

### Equipment

A combination of cart-based bedside ultrasound machines and handheld machines were used throughout the curriculum. The brand of ultrasound machine varied based on the location of the training. Having different equipment, although not required to implement this curriculum, augmented the curriculum by allowing learners the ability to gain experience with multiple types of POCUS machines.

### Quality assurance and tracking numbers

Ultrasound examinations performed in the clinical setting for educational reasons and submitted through the ultrasound workflow solution (QPath, Telexy Healthcare, Inc., Maple Ridge, BC, CA) were quality assured by an ultrasound division faculty. After acquiring images, each learner filled out an exam defined worksheet explaining indication, findings and interpretation. QA included general feedback regarding image acquisition and interpretation, binary agreement with the findings and interpterion of the study. A five-point image quality review was utilized for all exams: 1) no recognizable structures, no objective data can be gathered, 2) minimally recognizable structures but insufficient for diagnosis, 3) minimal criteria met for diagnosis, recognizable structures but with some technical or other flaws, 4) minimal criteria met for diagnosis, all structures imaged well and diagnosis easily supported, 5) minimal criteria met for diagnosis, all structures imaged with excellent image quality and diagnosis completely supported. Only examinations meeting pre-defined criteria for image acquisition by exam type and correct interpretation were counted toward credentialing, see Table 2.

**Table 2:** Pre-defined criteria for image acquisition by exam type for basic credentialing

E-FAST	RUQ: diaphragm, Morison’s pouch and caudal liver tip LUQ: diaphragm and spleen Cardiac: subxiphoid or parasternal long axis Pelvis: 2 views (transverse and sagittal) with bladder in view Lung: visualize pleural line of anterior chest on left and right with decreased depth
Aorta	Proximal short axis with celiac artery or SMA in view Proximal long axis with spine in view Distal short axis Distal long axis with spine in view Bifurcation (video sweep) Measurements of outside wall to outside wall
Cardiac	Parasternal long axis Parasternal short axis Apical 4 chamber Subxiphoid IVC Minimum 3 satisfactory of the 5 views required, ideally all for best interpretation
Transabdominal first trimester obstetric	Long axis: Uterus and ladder in view Short axis Depth must be adequate to evaluate cul-de-sac for free fluid Left adnexa Right adnexa
Soft tissue	Affected site in 2 orthogonal planes Measure size and depth
Thoracic	Zones 1–4 right Zones 1–4 left Costophrenic angles

*E-FAST* extended focused sonography in trauma, *IVC* inferior vena cava, *LUQ* left upper quadrant, *RUQ* right upper quadrant, *SMA* superior mesenteric artery

If the ultrasound examination was done in an educational setting (i.e. a workshop, “Drop in and sound” session) with an ultrasound division member overseeing the examination then real-time feedback was given. These numbers were tracked, signed off by an ultrasound faculty member and submitted on paper at the end of the session.

Faculty scan counts, whether submitted from educational sessions or through the workflow solution, for each modality were recorded in a Microsoft Excel (Microsoft Corp., Redmond, WA) file and distributed to faculty quarterly.

### Assessments

Knowledge and skill with POCUS were assessed in multiple ways throughout training and included post-tests, post-workshop objective standardized clinical examination (OSCE), and QA on images independently obtained by faculty after their initial training. A pre-test was given to every faculty before an ultrasound workshop to assess baseline knowledge. During the initial implementation, the only post-test administered was after the minimum of 16 h of CME and 100 QA-approved scans were completed in 2018. Starting in 2019, a post-test was given after each basic or advanced workshop in addition to the post-tests given to complete Basic Certification. The pre- and post-tests given before or after each workshop included five questions from each topic being covered during the workshop. The test given to complete Basic Certification included ten questions about each topic (physics, aorta, cardiac, E-FAST, OB) and had a 70% minimum passing grade. The tests given at the end of Basic Certification were closed-book but learners had unlimited attempts to achieve a passing score. See Additional file 1: Post-survey, Additional file 2: Post-survey and Additional file 3: Pre-tests and Additional file 4: Post-tests.

Beginning in 2019, each ultrasound training session concluded with an OSCE. These exams were completed using standardized patients and administered by ultrasound trained faculty. They were tailored to the topics covered during the respective workshop. Learners were asked to demonstrate correct machine usage including entering patient information, selecting the correct scanning modality, choosing the correct probe, and demonstrating correct positioning of the indicator. They were then asked to acquire representative images for each scanning modality taught. The ultrasound faculty graded their ability to acquire images and recorded the quality of the image obtained. Learners were also asked to identify relevant anatomy and their ability to do so was recorded. See Additional file 5: Post-OSCE. No assistance was given by the ultrasound faculty until after the OSCE had concluded (Additional file 6)*.*

### Coordination with department initiatives

In 2018, a new financial incentive model was introduced by the Department of Emergency Medicine to incentivize physician behavior in the department. This new financial model was broadly deployed across the entire physician practice group and paid out according to pre-approved incentives that were chosen by the department chair and approved by senior leaders in the department and the physician practice group. The first year of the plan included a POCUS training incentive, among two other department incentives, with the goal of achieving Basic Certification in accordance with ACEP Ultrasound Guidelines [1]. We found a substantial increase in the number of POCUS examinations being performed beginning after the incentive was implemented. In 2017, there was a total of 179 quality assured scans performed and this increased to 2916 in 2018 and 4879 in 2019. Due to the success of this incentive program, two additional POCUS incentives were created in the subsequent years to help faculty achieve Global Certification. Refer to Table 3 for maximum POCUS payout by year for each individual faculty beginning in 2018.

**Table 3:** POCUS incentive payout by year for each faculty

Year	POCUS incentive ($)
2018	$6453
2019	Academic—$7008 Community—$9729
2020	$6500

## Future directions

A significant amount of time and resources were dedicated to implement this curriculum. This included an incentive for faculty to complete the curriculum, as well as dedicated full-time equivalent (FTE) for instructors to plan and run CME events, QA submitted scans, track numbers and teach at hands-on session. It is unknown if this training pathway will result in POCUS integration into clinical practice. Appropriate POCUS use with subsequent documentation in the electronic medical record is one long term measure of success. Measuring this outcome is ongoing and will be a focus of future study. Further incentives are planned to reinforce the process of clinical documentation, which requires additional steps to capture correct patient data and complete POCUS documentation in a separate workflow system. These additional steps can be cumbersome and challenging to perform in a busy practice setting.

## Conclusion

In this paper, we describe a successful approach to credentialing EM faculty in POCUS, in both an academic and community setting. Having a standardized approach for achieving competency and credentialing in POCUS is important. This paper can serve as a reference for other departments going through the same process.

## Supplementary Information

**Additional file 1. **Pre-survey.

**Additional file 2. **Post-survey.

**Additional file 3. **Pre-test.

**Additional file 4.** Post-test.

**Additional file 5. **Post OSCE.

**Additional file 6. **Pre- and post-soft tissue and thoracic test (for the pediatric faculty).

## Data Availability

Not applicable.
