# Supplementary material for: Design and implementation of a basic and global point of care ultrasound (POCUS) certification curriculum for emergency medicine faculty
Source: Ultrasound J. 2022 Feb 19;14:10. doi: 10.1186/s13089-022-00260-y (PMC8858359; doi:10.1186/s13089-022-00260-y)
Supplement: Supplementary file 1 — Additional file 1. Pre-survey. [file 13089_2022_260_MOESM1_ESM.docx]

**Pre-Survey**

Name:

Date:

What year did you graduate residency?

Level of US experience:

Never touched a machine/none to very little

Some/used a couple times

Moderate/use a couple times per month

Large amount/completed a rotation or elective/use almost every shift

Other:

How many ultrasounds have you performed in the last 2 years?

0 1-10 11-25 26-50 51-100 >100

Barriers to use US (circle all that apply):

Takes too much time

Don’t know how to use the machine

Don’t know how to interpret images

Comfort level of personal skills

I don’t see utility of using it

Lack of machine availability

Too busy/time consuming

Don’t know how to use/submit on QPath

Don’t know when to use the ultrasound machine

None

Other (fill in):

**Rate on a scale of 1-5, with 1 = strongly disagree, 3 = neutral, and 5 = strongly agree**

I feel comfortable using the ultrasound machine

1    2    3    4    5

I feel comfortable obtaining US images

1    2    3    4    5

I feel comfortable interpreting US images

1    2    3    4    5

I feel comfortable incorporating US into clinical practice

1    2    3    4    5

I feel comfortable knowing the indications for point-of-care ultrasound (POCUS)

1    2    3    4    5
