# Supplementary material for: Design and implementation of a basic and global point of care ultrasound (POCUS) certification curriculum for emergency medicine faculty
Source: Ultrasound J. 2022 Feb 19;14:10. doi: 10.1186/s13089-022-00260-y (PMC8858359; doi:10.1186/s13089-022-00260-y)
Supplement: Supplementary file 3 — Additional file 3. Pre-test. [file 13089_2022_260_MOESM3_ESM.docx]

**PRE-Test**

1. Sound __________________.

A. is a series of compressions and rarefactions

B. does not require media for transmission

C. travels best through air

D. carries both matter and energy

2. The propagation speed of sound in soft tissue is:

A. 330 m/sec

B. 1450 m/sec

C. 1540 m/sec

D. >2000 m/sec

3. What is the proper order of sound propagation speed in the following media?

A. soft tissue < fat < air < bone

B. bone < air < fat< soft tissue

C. fat < air < soft tissue < bone

D. air < fat < soft tissue < bone

4. Which statement is correct?

A. Higher frequency probes give better resolution than lower frequency probes but have greater attenuation.

B. Higher frequency probes give less resolution than lower frequency probes.

C. Lower frequency probes have more attenuation than higher frequency probes.

D. Probe frequency does not affect resolution or attenuation.

5. The primary component of sound attenuation in tissue is:

A. Scatter

B. Absorption

C. Reflection

D. Refraction

6. The earliest definitive sign of intrauterine pregnancy (IUP) is the presence of a:

1. Gestational sac
2. Fetal pole inside a gestational sac
3. Yolk sac inside a gestational sac
4. Fetal pole with a heartbeat inside a gestational sac

7. In a pregnant patient with vaginal bleeding and reassuring fetal heart tones on ultrasound, what is the risk of spontaneous abortion for this patient?

1. 50%
2. 25%
3. 10%
4. 5%

8. The fetal heart rate is most safely measured with:

1. B mode
2. M mode
3. Power Doppler
4. Color Doppler

9. Which of the following should NOT be used to determine gestational age:

A. Gestational sac diameter

B. Crown-rump length

C. Biparietal diameter

D. Femur length

10. If a pregnant patient is unstable, and no IUP is seen on your OB ultrasound which exam should be performed next?

A. Aorta

B. Cardiac

C. FAST

D. CT abdomen and pelvis

11. Any abdominal aorta measurement greater than _____ is considered aneurysmal

A. 2.5 cm

B. 3 cm

C. 3.5 cm

D. 4 cm

12. The aorta should be measured in the ___ axis from ___ to ___.

A. Sagittal (long), inside, inside

B. Sagittal (long), outside, outside

C. Transverse (short), outside, outside

D. Transverse (short), inside, outside

13. True or False: Lack of intraperitoneal free fluid on ultrasound rules out rupture of aortic aneurysm in an unstable patient?

A. True

B. False

14. Which view is not required for a thorough Aorta Ultrasound study?

A. Proximal Transverse

B. Proximal Longitudinal

C. Distal Transverse

D. Distal Longitudinal

E. Bifurcation

F. None of the above

15. True or False: Ultrasound can rule out dissection when the aorta is adequately imaged.

A. True

B. False

16. Which view is not part of the basic ED cardiac ultrasound examination?

A. Parasternal Long Axis

B. Parasternal Short Axis

C. Apical 5 chamber

D. Subxiphoid

17. If you are having trouble imaging the heart, the patient should be

A. Placed in left lateral decubitus

B. Placed in right lateral decubitus

C. Sitting at 45 degrees

D. Placed in Trendelenburg

18. How do you tell the difference between pleural and pericardial effusion?

A. Pleural effusion is posterior or lateral to descending aorta

B. Pleural effusion is medial or anterior to descending aorta

C. Pleural effusion is anterior to the heart

D. Pleural effusion is more echogenic than a pericardial effusion

19. What is the most sensitive finding for tamponade?

A. Right ventricular diastolic collapse

B. Left ventricular diastolic collapse

C. Left atrial diastolic collapse

D. Right atrial diastolic collapse

20. The best view to compare sizes of the ventricles is the

A. Apical short axis

B. Apical 4-chamber

C. Parasternal long axis

D. Parasternal short axis

21. What is the approximate sensitivity/specificity of FAST for detecting intra-abdominal free fluid?

A. 75% / 95%

B. 50% / 50%

C. 20% / 80%

D. 95% / 95%

22. Where do you look for free fluid in the FAST exam?

A. Pericardial sac, Morrison’s pouch, splenorenal recess, retrovesicular/pouch of Douglas

B. Around liver tip, between liver and diaphragm, between spleen and diaphragm, in long and short axis of the bladder

C. All of the above

23. True or False: The EFAST is more sensitive and just as specific as portable chest X-ray for detection of pneumothorax.

A. True

B. False

24. True or False: a negative FAST exam allows you to rule out significant intra-abdominal solid organ injury.

A. True

B. False

25. You will see fluid first in the LUQ:

A. Between Diaphragm and Spleen

B. Within the Spleen

C. Between Spleen and Kidney

D. Within the Kidney

ANSWER KEY:

1. A
2. C
3. D
4. A
5. B
6. C
7. D
8. B
9. A
10. C
11. B
12. C
13. B
14. F
15. B
16. C
17. A
18. A
19. D
20. B
21. A
22. C
23. A
24. B
25. A
