# Supplementary material for: Design and implementation of a basic and global point of care ultrasound (POCUS) certification curriculum for emergency medicine faculty
Source: Ultrasound J. 2022 Feb 19;14:10. doi: 10.1186/s13089-022-00260-y (PMC8858359; doi:10.1186/s13089-022-00260-y)
Supplement: Supplementary file 4 — Additional file 4. Post-test. [file 13089_2022_260_MOESM4_ESM.docx]

**POST-Test**

1. The propagation speed of sound in soft tissue is:

A. 330 m/sec

B. 1450 m/sec

C. 1540 m/sec

D. 1875 m/sec

E. > 2000 m/sec

2. You perform an EFAST exam on your trauma patient. What findings are seen in the RUQ view shown below?


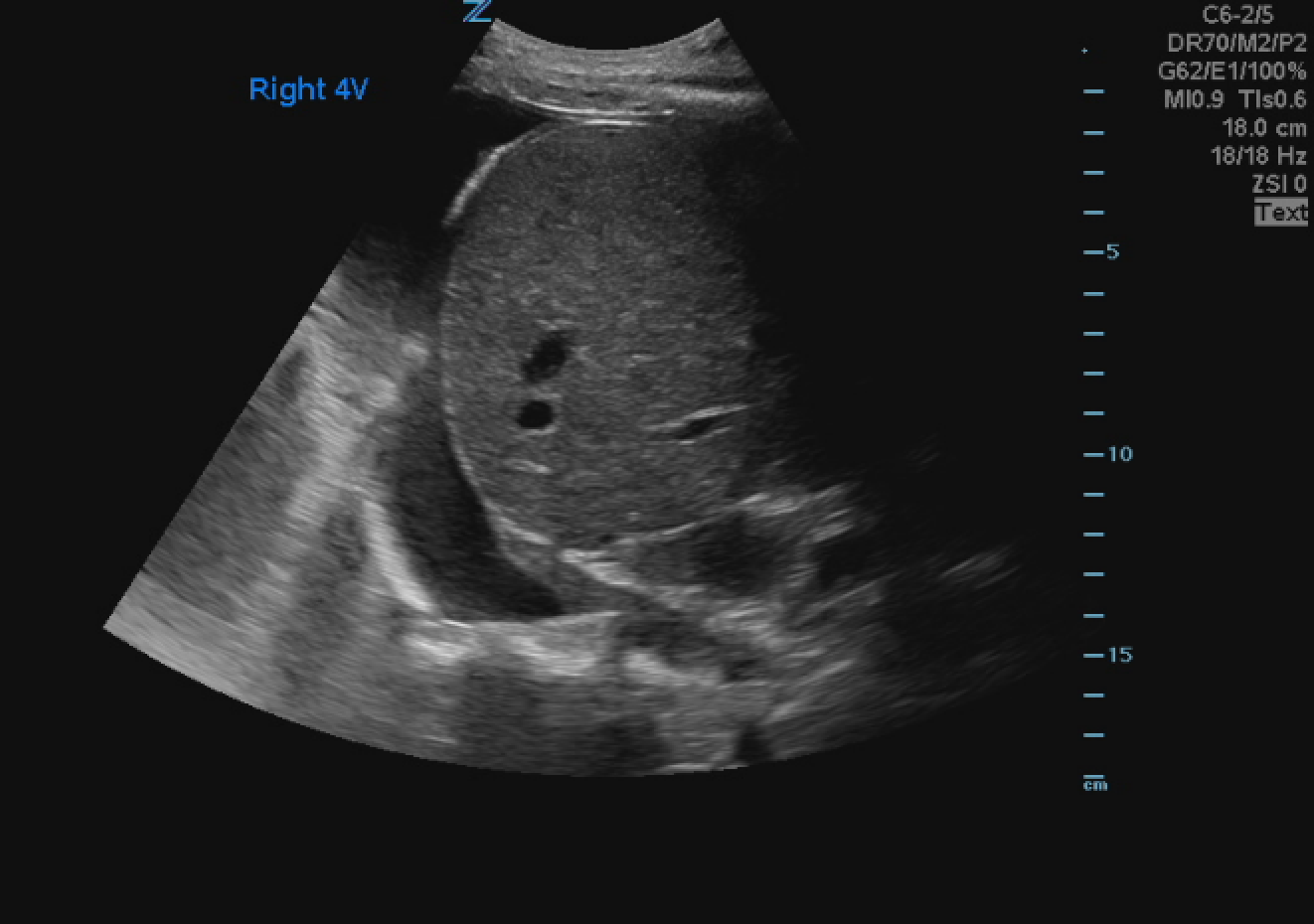


A. Liver laceration

B. Traumatic diaphragmatic hernia

C. Hemothorax

D. Pulmonary contusion

E. Pneumothorax

3. Your patient with abdominal pain is found to have a quantitative b-HCG level of 16,000. Your bedside ultrasound demonstrates the following. Based on this image, what is the next best option?


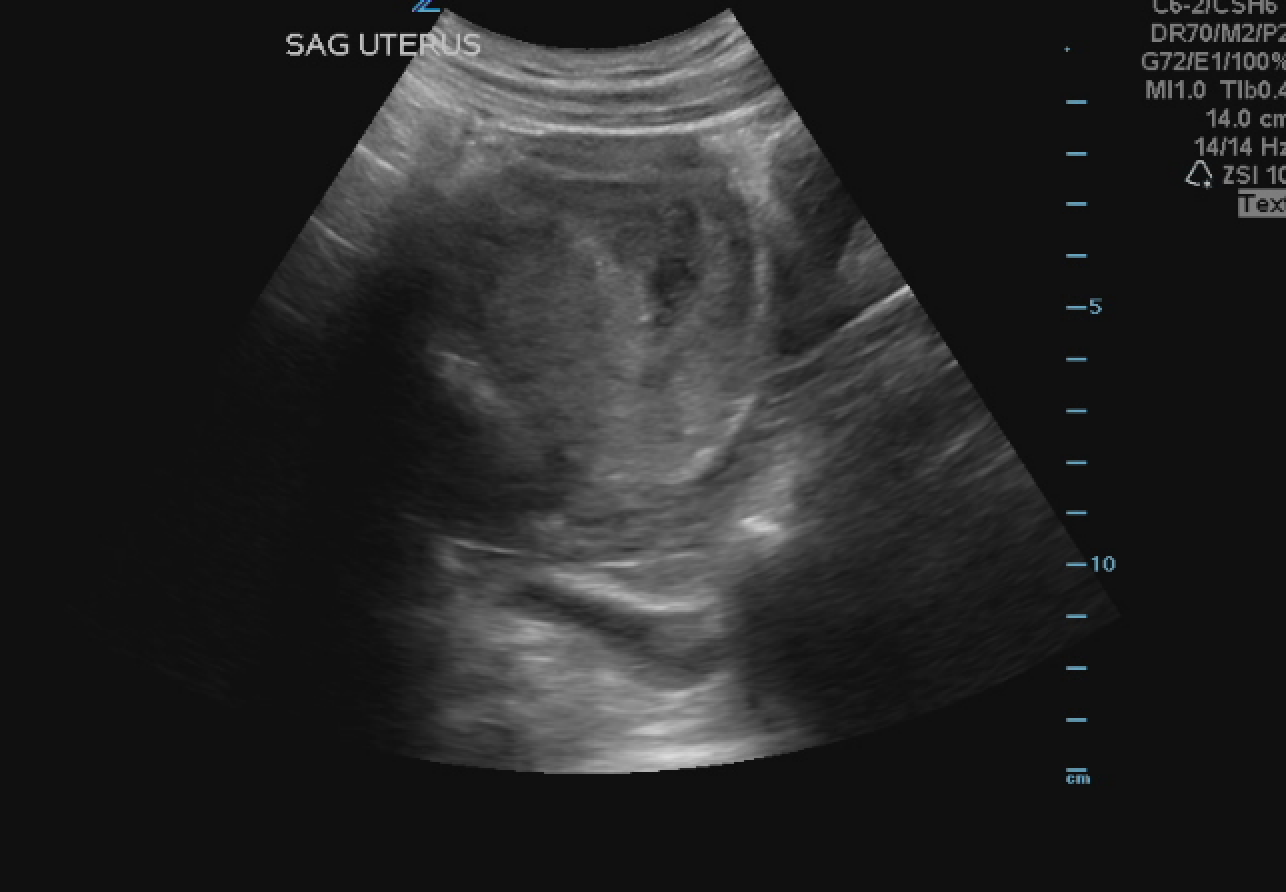


A. Release for 72 hour follow-up with OB

B. MRI abdomen to evaluate for appendicitis in pregnant patient

C. Obtain cath urine

D. OB consult

E. Perform aorta ultrasound

4. Which of the following are indications for performing a focused bedside cardiac ultrasound exam:

A. To rule out a subsegmental pulmonary embolus

B. To rule out cardiac tamponade in a patient with a known large pericardial effusion

C. To rule out focal wall motion abnormalities in a patient with chest pain and new T wave inversions on their ECG

D. To rule out hemopericardium in a patient with a stab wound to the upper abdomen

E. None of the above

5. Which statement is correct?

A. Higher frequency probes give better resolution than lower frequency probes, but have greater attenuation.

B. Higher frequency probes give less resolution than lower frequency probes.

C. Lower frequency probes have more attenuation than higher frequency probes.

D. Probe frequency does not affect resolution or attenuation.

E. None of the above

6. You perform a cardiac ultrasound on a patient with acute dyspnea and chest pain. What possible diagnosis best correlates with this image?


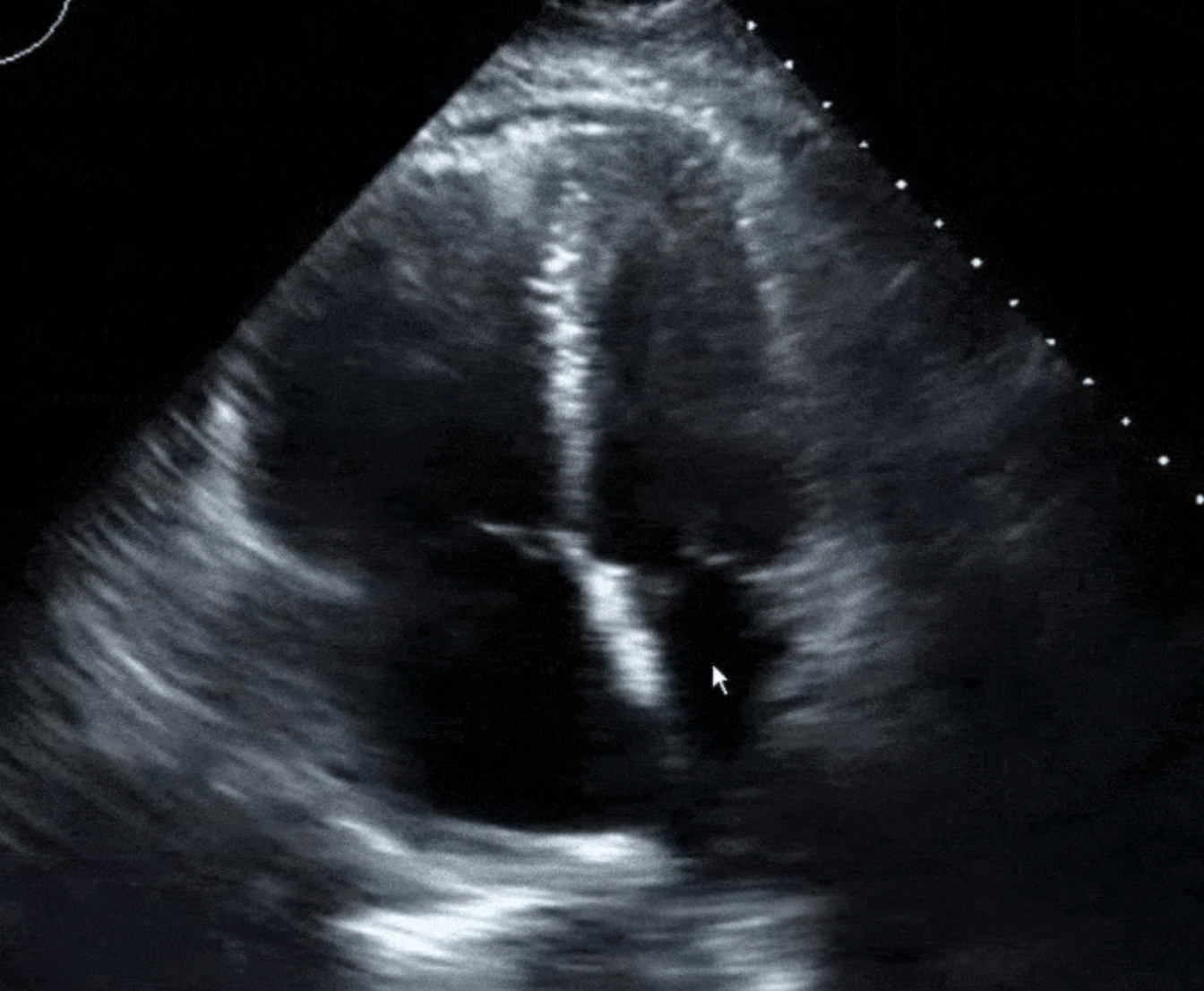


A. Right heart strain concerning for acute pulmonary embolism

B. Pericardial effusion with tamponade

C. Normal bedside cardiac imaging

D. Congestive heart failure

E. Subacute bacterial endocarditis

7. The earliest definitive sign of intrauterine pregnancy (IUP) is the presence of a:

A. Gestational sac

B. Fetal pole inside a gestational sac

C. Yolk sac inside a gestational sac

D. Fetal pole with a heartbeat inside a gestational sac

E. All of the above

8. This cardiac ultrasound image is in full diastole. What answer best describes the abnormality seen?


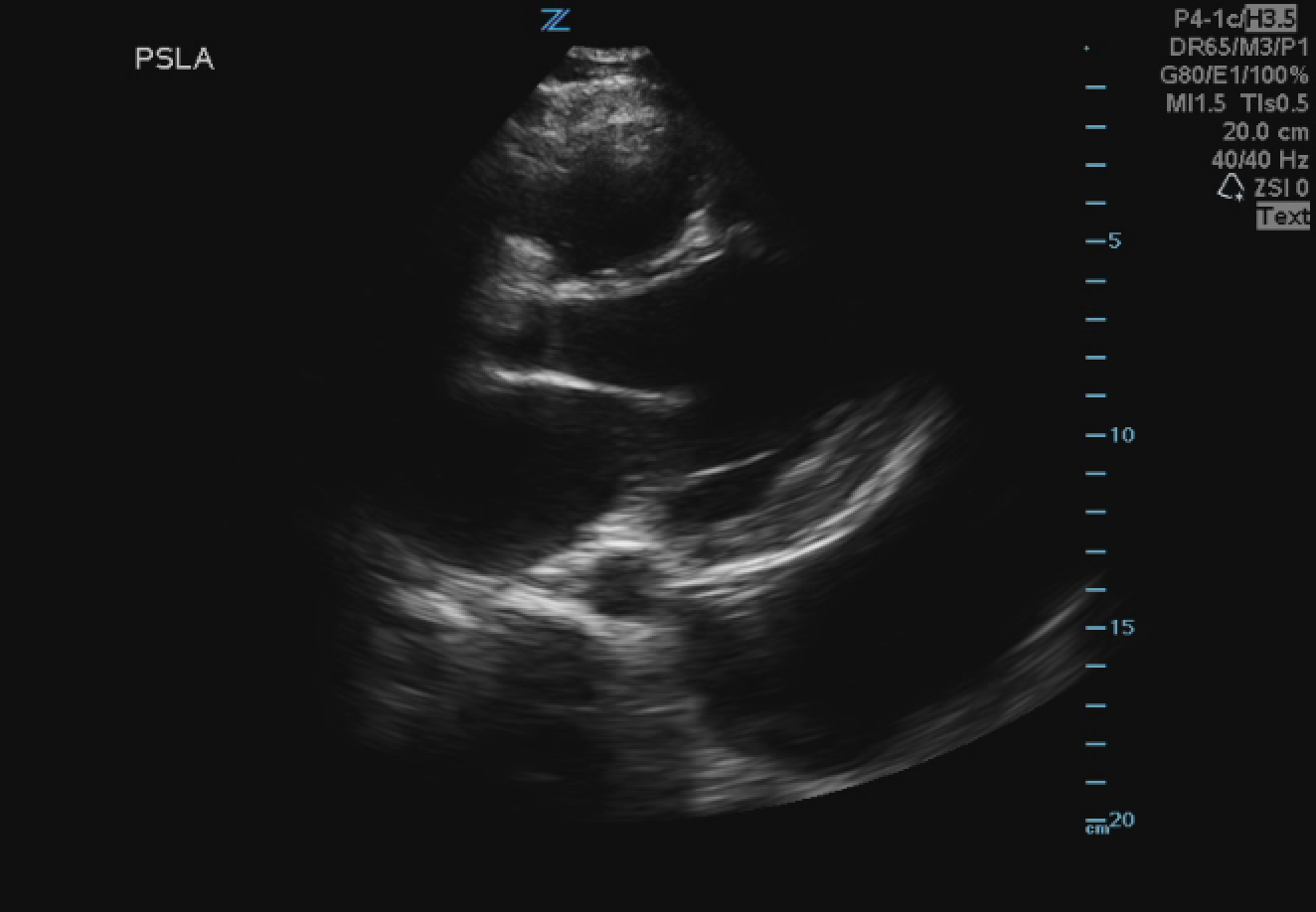


A. Pericardial effusion without tamponade

B. Right heart strain

C. Abnormal left ventricular outflow tract

D. Large valvular vegetation

E. Increased end point septal separation suggestive of poor LV function

9. The fetal heart rate is most safely measured with:

1. B mode
2. M mode
3. Power Doppler
4. Color Doppler
5. Gestalt

10. Any abdominal aorta measurement greater than _____ is considered aneurysmal

A. 2.5 cm

B. 3 cm

C. 3.5 cm

D. 4 cm

E. 4.5 cm

11. What is represented by the arrow?


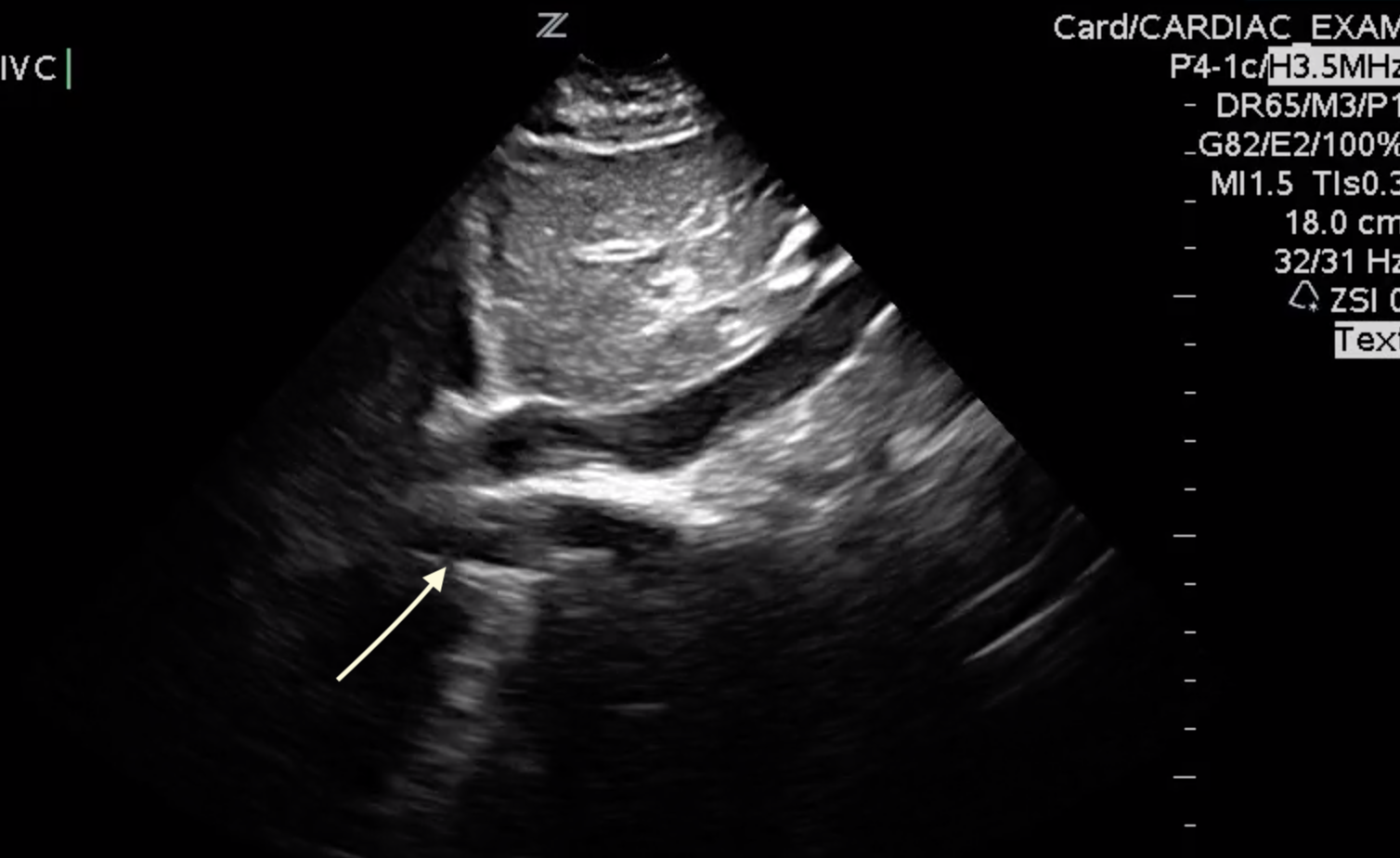


A. Congenital duplication of IVC

B. Speed displacement artifact

C. Refraction artifact

D. Both aorta and IVC seen in the same image

E. Mirror image artifact

12. As part of your EFAST exam, you obtain the image below. Your patient is awake and has a blood pressure of 80/palp, heart rate 140, and respiratory rate 30. Which of the choices is most appropriate?


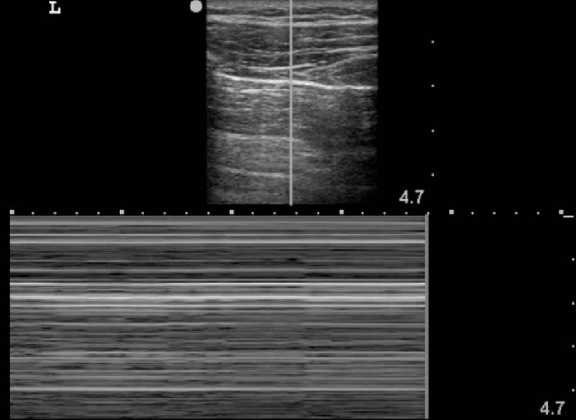


A. CT chest and abdomen with IV contrast

B. Place chest tube

C. Intubate

D. Order stat portable chest x-ray

E. Cardiovert

13. Which of the following is NOT an indication for emergency medicine OB/Gyn point-of-care ultrasound?

A. To rule-in an early intrauterine pregnancy

B. To rule-out an ectopic pregnancy

C. To assess for pelvic free fluid

D. To assess fetal heart rate and movement

E. All of the above are true indications

14. A 66yo male arrives to the ED with back pain that started abruptly last night as he was sitting on the couch. His blood pressure is 190/110. The rest of his vital signs are normal. His lumbar and thoracic back exam is normal. Is there an indication for performing a bedside ultrasound on this patient?

A. No. He is not hypotensive, therefore, there is no indication to evaluate for a ruptured abdominal aorta on a bedside ultrasound exam.

B. Yes. An abdominal aortic aneurysm is best seen when a patient is hypertensive.

C. Yes. An abdominal aortic aneurysm is a significant concern and a bedside ultrasound exam is indicated

D. No. He is not a good candidate for a bedside aortic ultrasound because he is in pain. His pain needs to be controlled before an adequate exam can be completed.

E. No. He needs a CT scan first.

15. True or False: Ultrasound can rule out dissection when the aorta is adequately imaged.

A. True

B. False

16. Your 23yo female patient is has a positive pregnancy test, and you do not see an intrauterine pregnancy on your bedside transabdominal ultrasound. What does this ultrasound image suggest?


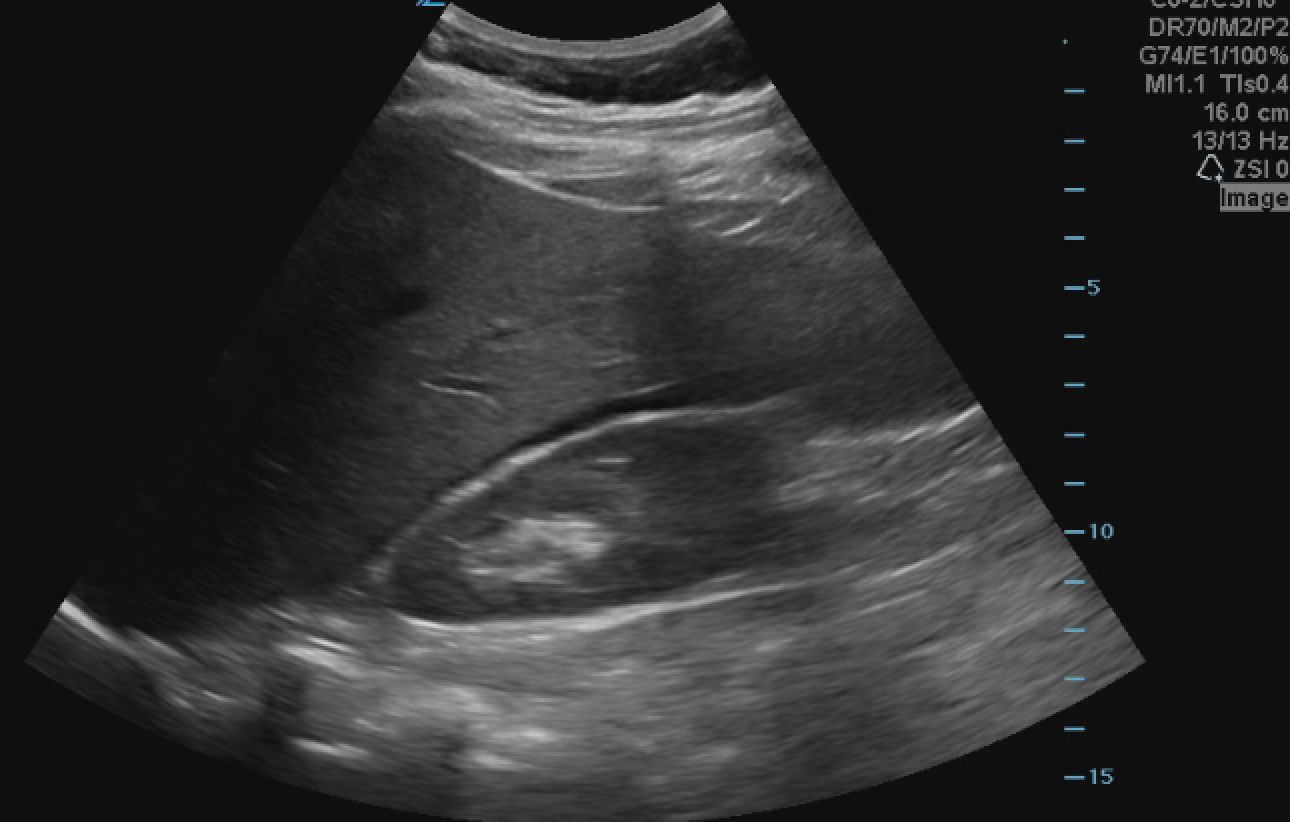


A. Patient has high probability of requiring surgical intervention for ruptured ectopic

B. Patient can be managed with methotrexate and serial hematocrits

C. It is likely there is Fitz-Hugh-Curtis syndrome with early pregnancy

D. False positive fluid in Morrison’s pouch is common in pregnancy

E. Normal image, patient should return in 48 hours for repeat testing

17. Which view is not part of the basic ED cardiac ultrasound examination?

A. Parasternal Long Axis

B. Parasternal Short Axis

C. Apical 5 chamber

D. Subxiphoid

E. Apical 4 chamber

18. What ultrasound artifact(s) are demonstrated in this image?


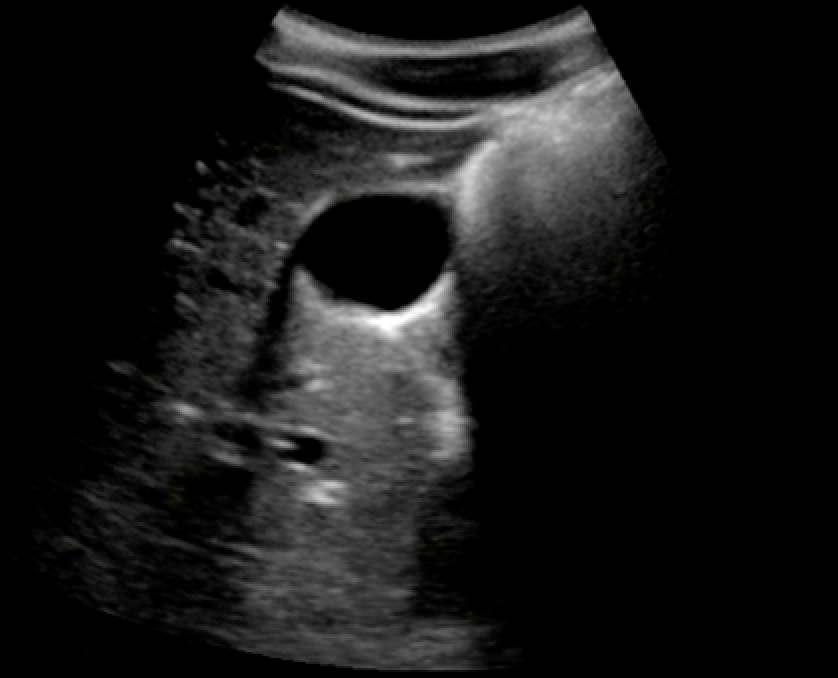


A. Posterior acoustic enhancement

B. Edge shadow

C. Mirror artifact

D. Both A and B

E. None of the above

19. Which is the least useful option in management of an unstable patient with this finding on bedside ultrasound?


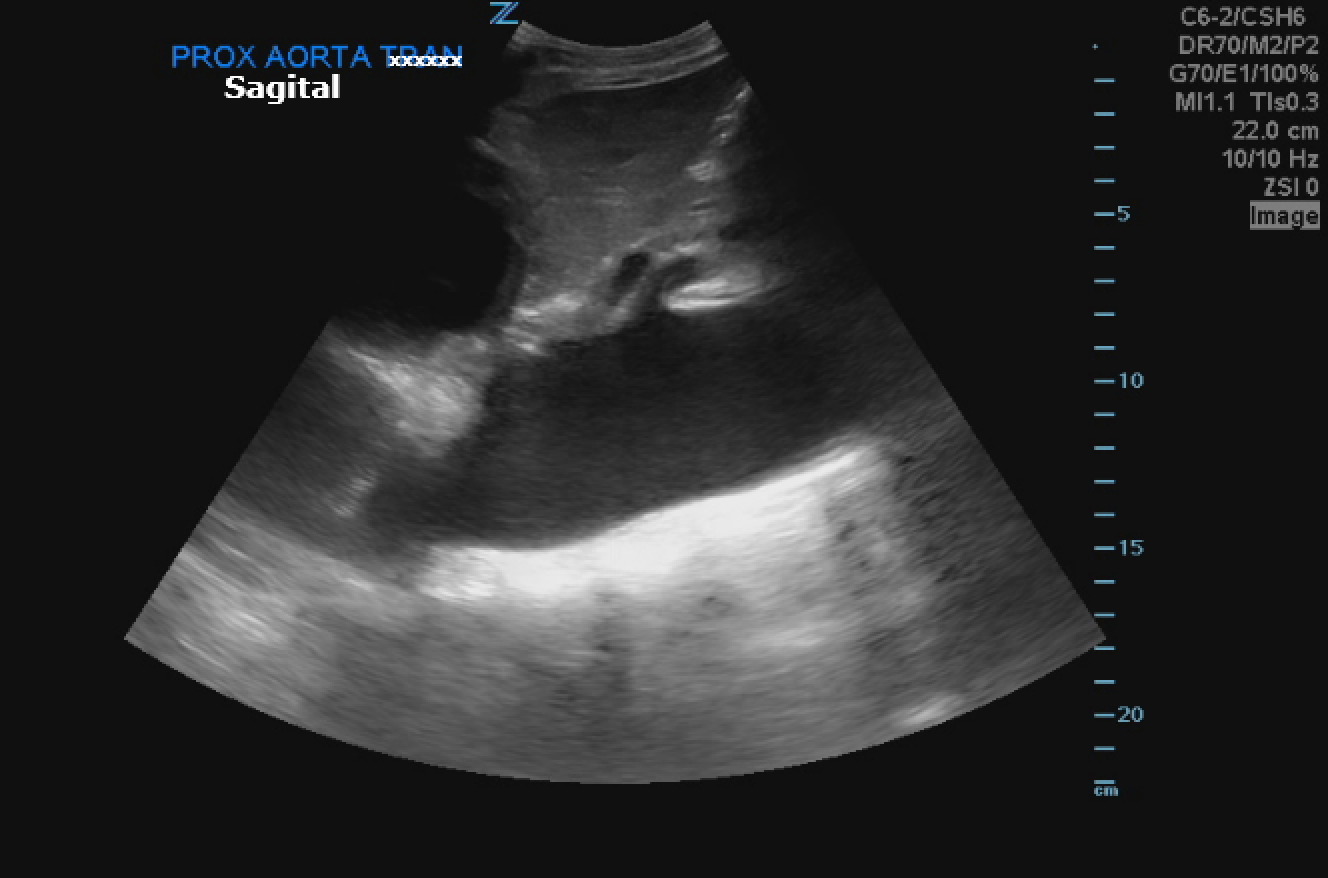


A. Obtain ankle-brachial index

B. Vascular surgical consultation

C. Type and crossmatch blood

D. Maintain 2 large bore IV’s

E. Transfuse uncrossmatched blood for hypotension

20. How do you tell the difference between pleural and pericardial effusion?

A. Pleural effusion is posterior or lateral to descending aorta on PSLA

B. Pleural effusion is medial or anterior to descending aorta on PSLA

C. Pleural effusion is anterior to the heart on PSLA

D. Pleural effusion is more echogenic than a pericardial effusion on PSLA

E. Pleural effusion cannot seen on PSLA

21. True or False: The EFAST is more sensitive and just as specific as portable chest X-ray for detection of pneumothorax.

A. True

B. False

22. You are suspicious of aortic dissection based on your patient’s clinical presentation. You perform a bedside aorta ultrasound and obtain this image. What is the most reasonable next step?


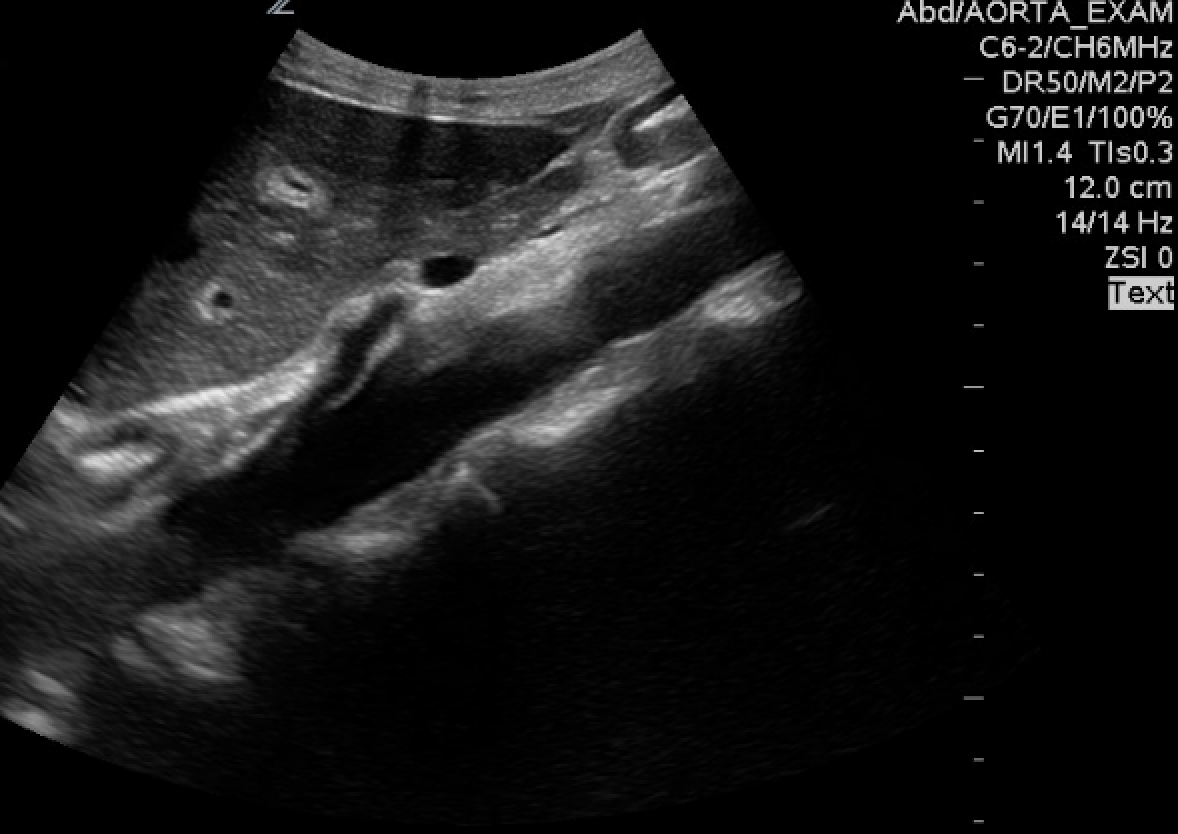


A. No dissection, discharge home

B. CTA, dissection protocol

C. D-dimer

D. Consult vascular surgery for a confirmed aortic dissection on POCUS

E. CXR

23. True or False: a negative EFAST exam allows you to rule out significant intra-abdominal solid organ injury.

A. True

B. False

24. When performing a EFAST exam, how can this image below be improved to better evaluate the pelvis for free fluid?


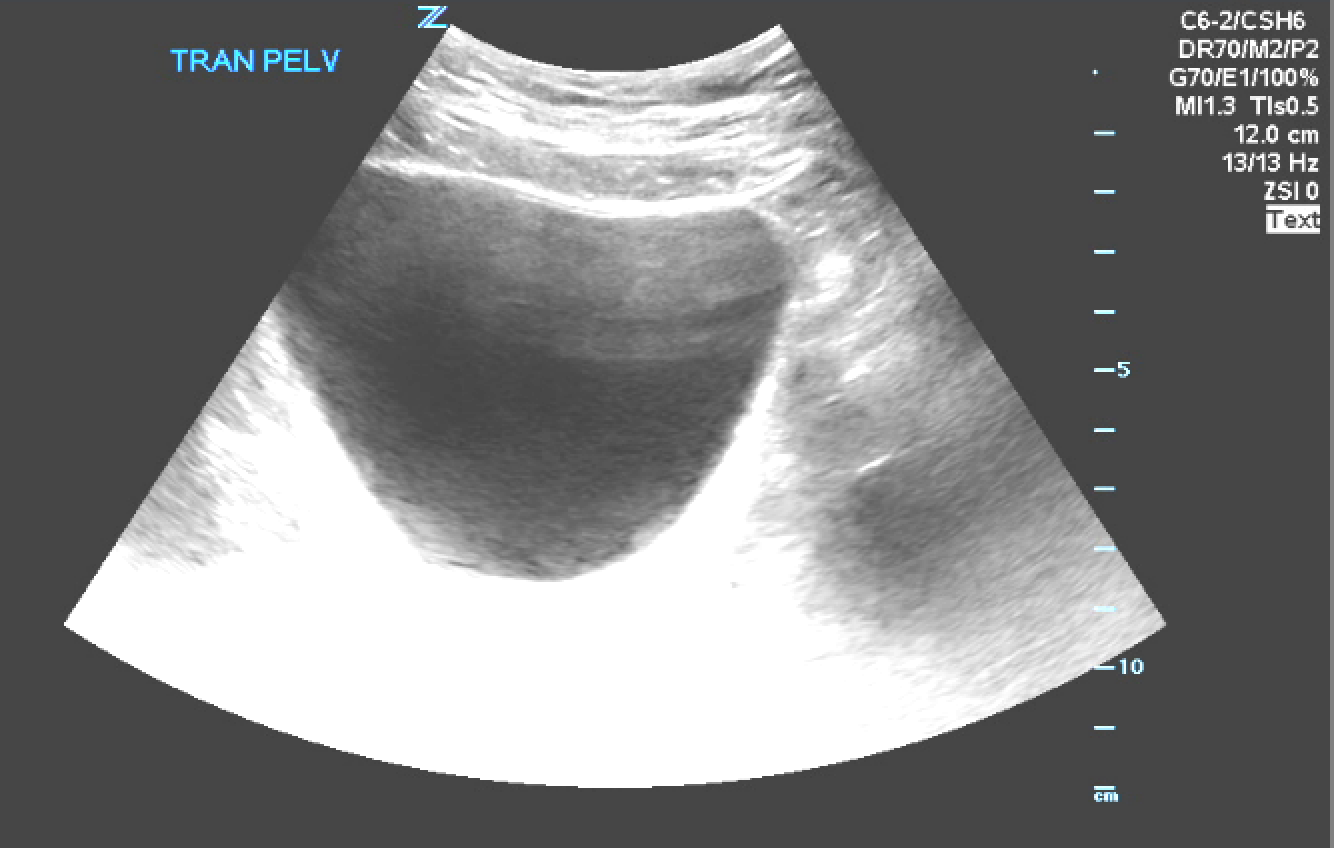


A. Reduce gain

B. Use power doppler mode

C. Change transducer

D. Increase depth

E. Nothing

25. All of the following are indications to perform an EFAST exam, except:

A. To evaluate for suspected ruptured ectopic in a hypotensive pregnant patient

B. To evaluate for bowel injury in a patient with penetrating abdominal injury

C. To evaluate for a hemothorax in a patient with stab wound to the chest

D. To evaluate for pneumothorax in a patient with blunt chest trauma

E. All of the above are indications for performing an EFAST exam

**ANSWER KEY:**

1. C
2. C
3. D
4. D
5. A
6. A
7. C
8. E
9. B
10. B
11. E
12. B
13. B
14. C
15. B
16. A
17. C
18. D
19. A
20. A
21. A
22. B
23. B
24. A
25. B
