# Supplementary material for: Design and implementation of a basic and global point of care ultrasound (POCUS) certification curriculum for emergency medicine faculty
Source: Ultrasound J. 2022 Feb 19;14:10. doi: 10.1186/s13089-022-00260-y (PMC8858359; doi:10.1186/s13089-022-00260-y)
Supplement: Supplementary file 5 — Additional file 5. Post OSCE. [file 13089_2022_260_MOESM5_ESM.docx]

**Instructors:**

Please fill out sheet. Do not provide any assistance until the end of the assessment at which time you can provide additional help.

**Evaluation metrics:**

**0 = Not sufficient.** Unable to identify structures; unable to perform machine/probe function.

**1 = Okay, but needs improvement.** Able to identify some structures, but not all; or able to perform some of the machine/probe functions, but with difficulty.

**2 = Sufficient.** Able to identify all structures; able to fully perform machine/probe functions.

**Machine Settings:**

1. Selects correct exam type 1 2 3

2. Selects correct probe for exam type 1 2 3

3. Marker dot pointed in the correct direction 1 2 3

4. Maneuvers probe to find a window sufficient to identify structures 1 2 3

5. Maneuvers probe to eliminate shadowing from ribs (if applicable) 1 2 3 N/A

6. Adjust depth appropriately 1 2 3

7. Adjusts gain appropriately 1 2 3

8. Measures appropriately (correct placement of calipers, locates measure) 1 2 3 N/A

**Image Acquisition and Interpretation:**

1. Aorta

- Identify the proximal aorta in short axis 1 2 3
- Identify surrounding structures (IVC, SMA, vertebral body shadow) 1 2 3
- Describe how to measure the aorta 1 2 3

2. Cardiac

- Obtain a PSLA view 1 2 3
- Identify structures (LV, RV, Ao root, MV, LA, Thoracic Aorta, pericardial sac) 1 2 3
- Describe where a pericardial effusion would collect 1 2 3

3. FAST

- Obtain a RUQ to evaluate for free fluid in the abdomen 1 2 3
- Identify the following structures (liver, kidney, Morison’s pouch) 1 2 3
- Describe where free intraperitoneal fluid would collect 1 2 3
- Obtain a RUQ to evaluate for free fluid in the pleural space 1 2 3
- Identify the following structures (liver, diaphragm, pleural space) 1 2 3
- Describe where free pleural fluid would collect 1 2 3

4. Comments:
