# Supplementary material for: Design and implementation of a basic and global point of care ultrasound (POCUS) certification curriculum for emergency medicine faculty
Source: Ultrasound J. 2022 Feb 19;14:10. doi: 10.1186/s13089-022-00260-y (PMC8858359; doi:10.1186/s13089-022-00260-y)
Supplement: Supplementary file 6 — Additional file 6. Pre- and post-soft tissue and thoracic test (for the pediatric faculty). [file 13089_2022_260_MOESM6_ESM.docx]

**Pre and Post Soft Tissue and Thoracic Ultrasound Test**

1. The appearance of intracellular edema as hypoechoic or anechoic strands which randomly traverse the subcutaneous fat can be referred to as:

A. Fatty Abscess

B. Cobblestoning

C. Necrotizing fasciitis

D. Hydroliposis

E. Lipoma

2. All of the following appear as hyperechoic on soft tissue ultrasonography EXCEPT:

A. Wood

B. Cyst

C. Glass

D. Plastic

E. Metal

3. Which of the following type of foreign body will have a characteristic “reverberation” artifact?

A. Wood

B. Cyst

C. Glass

D. Plastic

E. Metal

4. Which of the following can help to distinguish normal or reactive lymph nodes from an abscess?

A. Lymph nodes may have septations with ring down artifacts

B. Lymph nodes will have a “swirl” sign with gentle pressure

C. Lymph nodes will have hilar vascularity

D. More than 1 anechoic cavity is usually indicative of a lymph node

5. True or False: Necrotizing fasciitis can have a sonographic appearance similar to cellulitis.

A. True

B. False

6. Studies show that POCUS for soft tissue infection is superior to clinical examination alone with a sensitivity of

A. 40% vs. 50%, respectively

B. 98% vs. 86%, respectively

C. 85% vs. 80%, respectively

D. 75% vs. 65% respectively

7. A “halo sign” surrounding a foreign body on ultrasound is most commonly indicative of:

A. Reactive lymphadenopathy

B. Edema, granulation tissue or abscess

C. A foreign body within a blood vessel

D. Multiple foreign bodies in soft tissue

8. Which of the following is a characteristic sonographic finding of an abscess?

A. Ranges from anechoic to irregularly hyperechoic, internal echoes

B. Ranges from round and generally well-defined to irregular, lobulated

C. Posterior acoustic enhancement may be your only sonographic finding

D. “Swirl” sign may be seen with gentle pressure

E. All the above

9. The most important anatomic landmark(s) to identify on ultrasound for planning an abscess incision and drainage or foreign body removal is (are):

A. The dermis

B. Bone

C. Muscle fascia

D. Arteries and veins

10. Classic findings of necrotizing fasciitis on ultrasound may include the following:

A. Free fluid at the fascial plane

B. Posterior acoustic shadow

C. Commonly associated with a foreign body

D. Thinning of the subcutaneous layer

**Thoracic Ultrasound Quiz**

1. Which of the following is (are) an important reference point for identifying the pleural line and lung sliding?

1. Sternum
2. Image Depth
3. Ribs
4. Diaphragm

2. When evaluating for pneumothorax using ultrasound, what is the transition between normal lung sliding and static lung called?

1. Lung Point
2. Fluid Color sign
3. Lung Pulse
4. Lung Slide

3. Which of following is a characteristic of B-lines?

1. Discrete laser-like vertical hyperechoic reverberation artifacts
2. Arise from the pleural line
3. Extend to the bottom of the screen without fading
4. All the above

4. True or False: Lung ultrasound does not rule out pulmonary abnormalities that do not reach the pleura

1. True
2. False

5. All of the following choices have previously been described as indications for pediatric lung ultrasound EXCEPT:

1. Pneumonia
2. Malignant tumor
3. Bronchiolitis
4. Acute Respiratory Distress Syndrome

6. A meta-analysis in 2015 suggested a diagnostic accuracy of lung US for childhood pneumonia to be

1. Sensitivity 66%; Specificity 63%
2. Sensitivity 96%; Specificity 93%
3. Sensitivity 90%; Specificity 88%
4. Sensitivity 99%; Specificity 50%

7. Lung “hepatization” refers to

- 1. Isoechoic appearance of consolidation similar to the liver
  2. The costophrenic angle adjacent to the liver where an effusion is commonly seen
  3. Mirror artifact of the lung overlying the liver
  4. Diaphragmatic excursion of liver contents into the right lung base

8. Identify the curvilinear structure depicted by the arrow (white) in the abnormal image below:


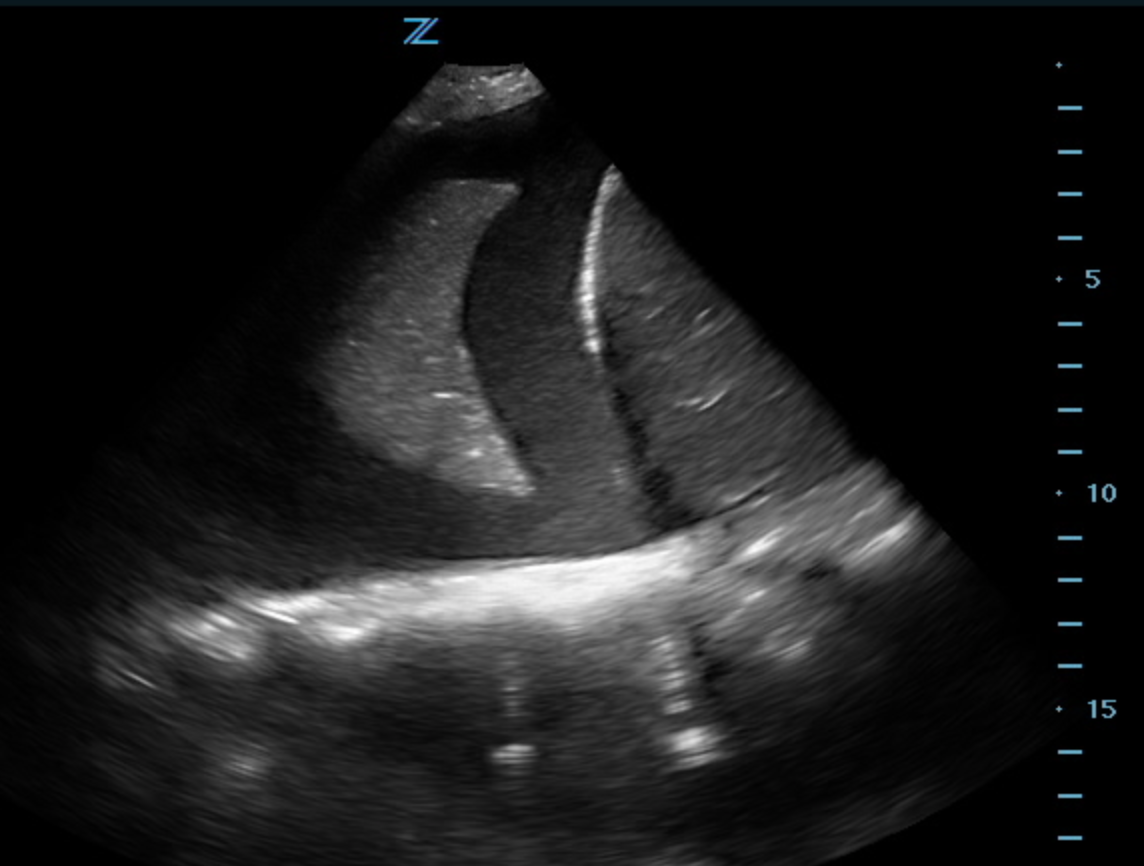


- 1. Liver edge
  2. Edge artifact
  3. Pleural effusion
  4. Diaphragm

9. Which of the following pathologic findings would have associated “B-lines”?

- 1. Asthma
  2. Pleural effusion
  3. Pneumothorax
  4. Bronchiolitis

10. Which of the following pathologic findings would have associated “A-lines”?

- 1. Pneumothorax
  2. Lung interstitial syndromes
  3. Pneumonia
  4. Pleural Effusion

**ANSWER KEY:**

**Soft Tissue**

1. B
2. B
3. E
4. C
5. A
6. B
7. B
8. D
9. D
10. A

**Thoracic**

1. C
2. A
3. D
4. A
5. B
6. B
7. A
8. D
9. D
10. A
